# Multiple morphophysiological responses of a tropical frog to urbanization conform to the pace-of-life syndrome

**DOI:** 10.1093/conphys/coad106

**Published:** 2024-01-27

**Authors:** Lilian Franco-Belussi, José Gonçalves de Oliveira Júnior, Javier Goldberg, Classius De Oliveira, Carlos E Fernandes, Diogo B Provete

**Affiliations:** Departamento de Ciências Biológicas, Universidade Estadual Paulista (UNESP), Instituto de Biociências, Letras e Ciências Exatas de São José do Rio Preto, São José do Rio Preto, São Paulo, 15054-000, Brazil; Instituto de Biociências, Universidade Federal de Mato Grosso do Sul, Campo Grande, Mato Grosso do Sul, 79002970, Brazil; Graduate Program in Animal Biology, Instituto de Biociências, Universidade Federal de Mato Grosso do Sul, Campo Grande, Mato Grosso do Sul, Brazil; Instituto de Diversidad y Ecología Animal - CONICET; Facultad de Ciencias Exactas, Físicas y Naturales, Universidad Nacional de Córdoba, Córdoba, Argentina; Departamento de Ciências Biológicas, Universidade Estadual Paulista (UNESP), Instituto de Biociências, Letras e Ciências Exatas de São José do Rio Preto, São José do Rio Preto, São Paulo, 15054-000, Brazil; Instituto de Biociências, Universidade Federal de Mato Grosso do Sul, Campo Grande, Mato Grosso do Sul, 79002970, Brazil; Instituto de Biociências, Universidade Federal de Mato Grosso do Sul, Campo Grande, Mato Grosso do Sul, 79002970, Brazil; Gothenburg Global Biodiversity Centre, Göteborg, Box 100, S 405 30, Sweden

**Keywords:** Cerrado, ecoimmunology, genotoxicity, germ cells, Pantanal, Scale mass index

## Abstract

The Pace-of-Life syndrome proposes that behavioural, physiological and immune characteristics vary along a slow-fast gradient. Urbanization poses several physiological challenges to organisms. However, little is known about how the health status of frogs is affected by urbanization in the Tropics, which have a faster and more recent urbanization than the northern hemisphere. Here, we analysed a suite of physiological variables that reflect whole organism health, reproduction, metabolic and circulatory physiology and leukocyte responses in *Leptodactylus podicipinus*. Specifically, we tested how leukocyte profile, erythrocyte morphometrics and germ cell density, as well as somatic indices and erythrocyte nuclear abnormalities differ throughout the adult life span between urban and rural populations. We used Phenotypic Trajectory Analysis to test the effect of age and site on each of the multivariate data sets; and a Generalised Linear Model to test the effect of site and age on nuclear abnormalities. Somatic indices, erythrocyte nuclear abnormalities, erythrocyte morphometrics and leukocyte profile differed between populations, but less so for germ cell density. We found a large effect of site on nuclear abnormalities, with urban frogs having twice as many abnormalities as rural frogs. Our results suggest that urban frogs have a faster pace of life, but the response of phenotypic compartments is not fully concerted.

## Teaser

Frogs that live in cities are subject to different environmental pressures. These animals tend to have a faster pace of life than wild animals. The rhythm of life of these animals causes changes in different systems. Somatic indices and blood characteristics differ between populations, but less so for germ cell composition.

## Introduction

The Pace-of-life syndrome (PoLS) refers to the concerted change in several life-history traits, including behaviour, physiology and immune response (Ricklefs and Wikelski, 2002; Réale *et al.,* 2010). Under this perspective of life history evolution, populations would be able to change their traits in response to environmental variation. However, changes along one axis would constrain changes along other axes due to inherent trade-offs (Hutchings, 2021). For example, maximizing survival might imply a decrease in reproductive capacity. While evidence for the existence of such correlation between traits is mixed (Royauté *et al.,* 2018), this framework is still useful for understanding how phenotypes change in response to environmental alterations, such as urbanization (e.g. Brans and De Meester, 2018). As a result, populations can be classified along a slow-fast continuum of life history strategies (see also Levins, 1968). However, most studies addressing PoLS on vertebrates in the context of urbanization have primarily focused on birds in the Northern Hemisphere (Royauté *et al.,* 2018) or have only examined one or two axes of life history variation (e.g. either behaviour or physiology or host–parasite interaction). Consequently, little is known about how frogs respond to urbanization in the Tropics, which have a more recent and intense urbanization process than the northern hemisphere (Shackleton *et al.,* 2021). Additionally, few studies have used an integrative approach, examining multiple variables from different organismal characteristics (e.g. immune system, metabolism, reproductive system, whole organism) throughout the adult life span of a vertebrate to test hypothesis on phenotypic responses to land use change. This information is crucial to understand organismal responses to urbanization and develop better conservation programmes.

Somatic indexes can provide a non-intrusive method to assess an organism health status, metabolism and fitness. For example, body condition is usually not only related to energetic reserves or the amount of lipid accumulated (Peig and Green, 2009) but also a potential mediator of immunity. Individuals with reduced body condition might allocate energy to less costly immune responses (Schoenle *et al.,* 2018). The Scaled Mass Index (Peig and Green, 2010) has been widely used to measure the body condition in a variety of taxa, especially frogs (MacCracken and Stebbings, 2012; Brodeur *et al.,* 2020), because it incorporates non-linear growth, it is independent of body size, and is correlated with the amount of lipids stored. Additionally, this index has successfully been used to detect differences in amphibian body condition following habitat alterations, such as urbanization (Iglesias-Carrasco *et al.,* 2017; Cogalniceanu *et al.,* 2021). Therefore, body condition offers an easy and rapid way to measure individual health status, enabling early detection of habitat degradation and population decline. Similarly, other somatic indexes, such as the gonadosomatic, hepatosomatic and splenosomatic measure the ratio between the mass of the respective organ and the body. The gonadosomatic index can provide information on reproductive output of an individual, reproductive strategy and population recruitment. The hepatosomatic index provides rough estimate about of metabolism, xenobiotics catabolism and energy storage (Chaves *et al.,* 2017). The splenosomatic index provides a rough estimate of the adaptive immune capacity of frogs (Rollins-Smith and Woodhams, 2011) (e.g. in terms of B lymphocyte production). Blood glucose can also provide an indirect clue on energetic basal metabolism (de Amaral *et al.,* 2022). Additionally, glucose varies seasonally (de Amaral *et al.,* 2022) and increases with environmental temperature in frogs (Tindal, 1956).

Urbanization can have significant effects on the immune cells of frogs. The increase in human development and infrastructure may increase the exposure of urban frogs to pollutants, pathogens, artificial light at night and changes in diet, all of which consistently alter their leukocyte profile (Davis *et al.,* 2008; Rollins-Smith and Woodhams, 2011). The maturation of the immune system also varies throughout the life span of an organism, with younger individuals prioritizing resistance over tolerance, while the opposite happens in older animals (Schoenle *et al.,* 2018). However, little is known about the impact of multiple stressors related to urban life (Ellis *et al.*, 2011; Schoenle *et al.,* 2018) and senescence on the immunological responses of wild vertebrates (Becker *et al.,* 2020) in the Global South.

The environment can have a significant impact on the leukocyte profile of wild vertebrates (Davis *et al.,* 2008). Exposure to anthropogenic stressors, such as temperature changes, pollutants and pathogens, can alter the number and activity of leukocytes in frogs (Rollins-Smith and Woodhams, 2011; Zhelev and Popgeorgiev, 2021). Specific leukocytes may increase in number or change their morphology in response to environmental changes, such as increasing in size or developing new surface proteins to better recognize and respond to new pathogens (Maceda-Veiga *et al.,* 2015; Zhelev and Popgeorgiev, 2021). The ratios between specific leukocytes can also provide valuable information on chronic stress levels, health and quality of animals (Davis *et al.,* 2008). For example, the neutrophil/lymphocyte ratio is often used as a marker for chronic stress, since they respond to environmental changes at a later stage, within 60 min or more after the exposure (Davis *et al.,* 2018). As such, leukocyte profiles provide insights into the functioning of both innate and adaptive immune responses, and their changes remain consistent and for longer periods than hormones (Davis *et al.,* 2018). This characteristic is particularly useful when assessing the impact of urban–rural contrasts, because land use changes usually involve non-linear alterations of multiple environmental variables over extended periods of time, instead of a single disturbance pulse (Liu *et al.,* 2020).

Erythrocytes can also provide insights into the physiological responses of organisms to environmental stressors. Changes in erythrocyte size, shape and density reflect differences in oxygen transport requirements, metabolism and immune function (Zhelev and Popgeorgiev, 2021). Differences in environmental conditions, such as pollution, altitude and diet, can affect erythrocyte morphology (Navas and Chaui-Berlinck, 2007). These changes may be linked to differences in metabolic rates and oxygen requirements during different life stages. For example, animals in urban areas may be exposed to higher levels of air and water pollution, which can result in smaller erythrocytes due to heavy metal pollution (Zhelev and Popgeorgiev, 2021). Additionally, temperature and body mass increase erythrocyte volume in tetrapods (Gillooly and Zenil-Ferguson, 2014), while active foraging correlates negatively with erythrocyte area in lizards (Penman *et al.,* 2022). Similarly, metabolic rate is negatively related with erythrocyte size in fish (Lay and Baldwin, 1999). Erythrocytes with smaller surface area-to-volume ratio deliver oxygen faster to tissues, while those with larger nucleus-to-cytoplasm ratio have high nuclear activity, which might indicate an increased protein synthesis (Lay and Baldwin, 1999). Xenobiotics found in urban environments may induce genotoxic effects and cause several types of nuclear abnormalities in erythrocytes (Cruz-Santiago *et al.,* 2022), including enucleated cells, micronuclei and immature erythrocytes. An increase in abnormal erythrocytes may hinder the ability of an organism to cope with environmental changes. However, most of the research relating erythrocyte nuclear abnormalities to contaminants has been conducted in agricultural or industrial areas. Nevertheless, this tool may be useful for assessing how urbanization affects organismal health.

Habitat alteration, like urbanization, has the potential to alter the reproductive capabilities of species (Anzaldua and Goldberg, 2020). Environmental stressors can disrupt the hormonal control of reproduction, reducing sperm and oocyte productions (Wingfield and Sapolsky, 2003). Although this topic has not been extensively studied in amphibians, some authors have shown that frogs exposed to anthropogenic noise, at ecologically relevant levels, had sperm count and viability significantly decreased compared with the control group (Kaiser *et al.,* 2015). Similarly, photoperiod alteration in urban landscapes due to artificial light at night can suppress spermatogenesis by decreasing spermatocytes (Biswas *et al.,* 1978; Touzot *et al.,* 2020). Conversely, Sinsch *et al.* (2007) found that urban male Green Toads (*Bufotes viridis*) had smaller size at similar ages, were younger at first reproduction, and had lower survival than females. However, some authors found no differences in reproductive investment between urban, suburban and rural populations of two frog species (Jennette et al., 2018).

All the aforementioned variables can be related to the axes of Pace of Life syndrome, specifically metabolism, immune response and reproduction (Réale *et al.,* 2010). Here, we tested how a suite of morphophysiological characteristics of rural and urban populations of the Pointedbelly Frog, *Leptodactylus podicipinus*, change throughout their adult life span. Specifically, we asked if the phenotypic trajectories of rural and urban populations converge or diverge, indicating different responses to environmental factors. We hypothesize that urban frogs will exhibit coordinated responses of variables that facilitate a faster life cycle, as compared to rural ones. For example, assuming that the urban environment is more stressful, we expect that selection will favour a shorter lifespan and earlier reproduction (Levins, 1968; Hutchings, 2021), which will trade-off with a reduced immune response (McKean and Lazzaro, 2011) and a higher proportion of nuclear abnormalities.

## Materials and Methods

### Animal model and study sites

We collected 10 calling adult males of the Pointedbelly Frog (*Leptodactylus podicipinus*) by active search in breeding sites at night at the UFMS Pantanal Field Station (−19.576699° S, −57.019158° W), Corumbá, Central Brazil in September 2018; and 11 adult males in three urban areas in Campo Grande (−20.49788° S, −54.59441° W), Mato Grosso do Sul, Central Brazil in September 2018 (reproductive season). We identified adult males by two secondary sexual traits: presence of vocal sacs and nuptial pads. The latter is an important trait to evaluate sexual maturity (Izzo *et al.,* 1982), because they are related to high testicular hormonal levels and can securely indicate reproductive activity in males (Rebouças *et al.,* 2018). We only collected males due to the sampling strategy employed, which used acoustic signals to locate individuals. Previous studies found negligible differences between sexes in terms of haematological parameters (Franco-Belussi *et al.,* 2022 and references cited therein).

**Figure 1:**
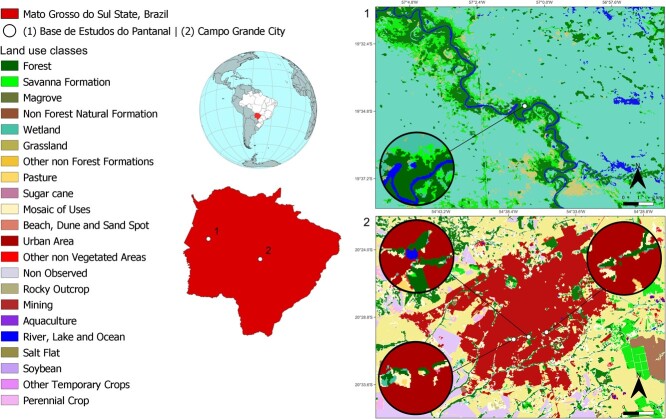
Map showing the two regions and percentage of building area and roads, a proxy commonly used to measure urbanization. Land cover data were obtained from MapBiomas v7.

These two regions are separated by 270 km (straight line). The percentage of building area and roads, a proxy commonly used to measure urbanization (Moll *et al.,* 2019; Szulkin *et al.,* 2020), in a 1-km buffer around the three sampling sites in Campo Grande varied between 67.72% and 83.16%, whereas that in Corumbá was 0% (Fig 1). These two regions also differ significantly in the amount of forest, grassland and Savanna both at the time of sampling and in 1985 (Supplementary Table S1). This confirms that the two regions have had distinct landscape compositions for at least 35 years. For the purposes of analysis, we grouped together individuals from the three urban sites. The land cover data were obtained from MapBiomas v7 at 30 m resolution (MapBiomas, 2023). The historical annual mean temperature ranges in Campo Grande are between 22 and 24°C, whereas that in Corumbá is 24°C to 26°C (de Oliveira Aparecido *et al.,* 2020). The annual rainfall ranges between 1500 and 2000 mm in Campo Grande, while in Corumbá it ranges between 1200 and 1500 (de Oliveira Aparecido *et al.,* 2020). Corumbá is in the Pantanal wetland and Campo Grande is in a highland Cerrado region. Climate in both areas is similar, which places them in the same Köppen-Geiger's Aw climate type (de Oliveira Aparecido *et al.,* 2020). Those two biomes have distinct ecological dynamics, with the Pantanal being regulated by flood pulses and strong seasonality in rainfall (Junk *et al.,* 1989), but not as much in temperature, whereas the Cerrado experiences strong temperature seasonality and a marked dry season. Ponds and lakes in the Pantanal rarely dry throughout the year, but those in the Cerrado usually do (Klink and Machado, 2005). The differences in ecological and climatic aspects between the two regions allow us to treat individuals from these regions as belonging to different populations, in the ecological sense.

This project was approved by the ethics committee for animal use of the Federal University of Mato Grosso do Sul [CEUA/UFMS 997/2018]. Collecting permits were provided by SISBIO/MMA to LFB [63297-1]. All procedures for the maintenance and handling of animals were carried out in accordance with the National Institute of Health Guide for Care and Use of Animals in the Laboratory.

### Body condition and somatic indices

Animals were anesthetized with 5% topical lidocaine on the ventral skin surface right before blood collection, which was made by cardiac puncture using syringes and needles with 3% ethylenediaminetetraacetic acid (EDTA) (Ranzani-Paiva *et al.,* 2013). Blood glucose (mg/dL^−1^) was estimated using portable digital glucometer (Accu-Chek Active). Subsequently, animals were euthanized with a higher dose of the same anaesthetic according to the procedures determined by the Colégio Brasileiro de Experimentação Animal (COBEA). Body size (Snout-Vent Length, mm) of animals was measured with a digital calliper. Whole animals and individual organs were weighed on a precision scale (to the nearest 0.001 g), and the testes, spleen and cardiac ventricle were removed to calculate somatic indices. Splenosomatic (ESI) and gonadosomatic (GSI) indexes and relative ventricular mass (RVM) were calculated as the ratio of (organ weight/animal weight) × 100.

We used the Scaled Mass Index (SMI) following (Peig and Green, 2009) to estimate the body condition of animals. The Major Axis Regression was done in the R package lmodel2 (Legendre, 2018). We used this index because it estimates changes in body mass scaled by body size (Peig and Green, 2010), allowing us to detect changes in body condition in response to environmental changes. It has also been commonly used in frogs (e.g. Brodeur *et al.*, 2020, MacCracken and Stebbings, 2012).

### Histological analysis of testes

The testes of 17 specimens (10 from rural and 7 from the urban area) were fixed in Metacarn (60 mL methanol, 30 mL chloroform and 10 mL acetic acid) for 3 hours, followed by histological processing for inclusion in Paraplast® (Leica). Sections of 3 μm were stained in haematoxylin and eosin. For the evaluation of germ cells, we analysed 10 photos per animal in the 40× objective. Structural volumetric density was used to estimate the percentage of each cell type at spermatogenesis stages: spermatogonia, spermatocytes, spermatids and loose spermatozoa in the lumen. Analysis was conducted using a grid with 252 intersections in Image J software (Rueden *et al.,* 2017). The density of each structure was calculated following the formula: Dve = (Ip × 100/252), where Ip are the positive intersections for the structure and 252 is the total number of intersections in the image (Freere and Weibel, 1967; Reid, 1980; Rocha *et al.,* 1997). Then, we calculated the density of each germ cell type in relation to the size of the locule to have the percentage of the locule occupied by oogonia, oocyte, spermatid and sperm.

### Estimating age with skeletochronology

To estimate the age of animals, we followed standard protocols of skeletochronology, with adaptations (Smirina, 1972; Castanet and Smirina, 1990). Briefly, we sliced the phalanges of the fourth toe to count the Lines of Arrested Growth (LAGs). The phalanges remained for five days in 10% EDTA for decalcification. Then, the tissue was dehydrated for embedding in Paraplast® (Leica). Sections of 10 μm were made in a microtome (Hyrax M25, Zeiss) and stained with haematoxylin and eosin, and then observed in a Zeiss optical microscope (Primo Star®) for the identification and counting of LAGs. Five sections per animal were analysed to evaluate distinct regions of the phalanges and confirm the number of LAGs. Thus, the age of individuals was estimated by counting LAGs according to Bionda *et al.* (2015) and Leskovar *et al.* (2011). Animals were further divided into three groups due to low sample size in each LAG: animals aged 1 to 2 years, 3 to 4 years and 5 to 6 years. Then, we converted groups into an ordered factor to represent age: young (1–2 LAGs), middle age (3–4 LAGs) and old animals (5–6 LAGs).

### Blood cell characteristics

For all subsequent analysis involving blood characteristics, we sampled blood from 21 individuals (10 from rural and 11 from urban sites). One blood smear per individual was made immediately after blood collection and stained with May–Grunwald–Giemsa–Wright (Tavares-Dias and Moraes, 2003). Then, we took several linear morphometric measurements from erythrocytes. Specifically, cell and nuclear volume, cell and nuclear area and nucleus/cytoplasm ratio in 50 cells per animal (see Franco-Belussi *et al.,* 2022 for more details on methods). To describe the leukocyte profile of the two populations, we counted 100 leukocytes in each blood smear to establish the percentage of each leukocyte found in frogs (differential leukocyte count): lymphocytes, basophils, neutrophils, monocytes, thrombocytes and eosinophils (Allender and Fry, 2008; Davis *et al.,* 2008). To count the total number of nuclear abnormalities, we evaluated 1000 erythrocyte nuclei per animal, recording all types of abnormalities (see Allender and Fry, 2008 for more details).

### Data analysis

Firstly, we divided our whole data set into four multivariate sets that describe a given biological process or body system and that had the same unit of measurement: germ cell density, leukocyte profile, erythrocyte morphometry and somatic indices. To summarize each of the four multivariate data sets, we first used Principal Component Analysis (PCA) separately for each of these sets of variables and retained all axes. Except for somatic indices, all other data were expressed as percentages or proportions. Compositional data are very common across ecology, behaviour and evolution (Pierotti and Martín-Fernaández, 2011; Greenacre, 2021), but poses a challenge to common statistical methods. In this case, we used a PCA for compositional data (Aitchison, 1983) in the R package compositions (van den Boogaart and Tolosana-Delgado, 2008).

Afterwards, we used Phenotypic Trajectory Analysis (PTA) to analyse each data set separately. This type of analysis is especially useful when one has an ordered predictor variable (e.g. age) whose levels are not considered as such in factorial models (Collyer and Adams, 2013). PTAs have been used in the context of Pace-of-Life syndromes recently (e.g. Brans and De Meester, 2018) to test the response of the multivariate phenotype on urban versus rural populations. Here, we were interested in understanding how each data set changed throughout the adult life span in response to rural versus urban environments. Before calculating trajectory attributes, we first built multivariate linear models (two-way MANOVAs) using all PCs for each data set as response variables, modelled as a function of site (factor with two levels) and age (ordered factor with three levels), including their interaction. Residual diagnostics was conducted by visually inspecting q-q plots and residual versus fitted (see Franco-Belussi *et al.,* 2023). No model violated assumptions.

**Table 1:** Results of the Multivariate Analysis of Variance (MANOVA) for testing the effect of site and age, and their interaction, separately on each multivariate data set

**Germ cells**	Df	SS	MS	R^2^	F	Z	*P*
Site	1	1.086	1.0857	0.00426	0.7567	0.0487	0.476
Age	2	4.368	2.1839	0.01713	1.5223	1.0075	0.164
Site:Age	2	14.177	7.0885	0.05562	4.9410	3.6211	**0.001**
Residuals	164	235.280	1.4346	0.92299			
Total	169	254.910					
**White cells**							
Site	1	6.927	6.9267	0.10347	2.6577	1.70340	**0.048**
Age	2	14.461	7.2303	0.21601	2.7742	2.29285	**0.007**
Site:Age	2	6.461	3.2306	0.09652	1.2395	0.61859	0.280
Residuals	15	39.095	2.6063	0.58400			
Site	20	66.943					
**Erythrocytes**							
Site	1	1326.7	1326.66	0.18050	239	11.9983	**0.001**
Age	2	9.9	4.93	0.00134	0.889	0.0450	0.472
Site:Age	2	218.3	109.17	0.02971	19.667	6.7688	**0.001**
Residuals	1044	5795.1	5.55	0.78845			
Total	1049	7350.0					
**Somatic indices**							
Site	1	21.897	21.8968	0.20854	5.7656	3.8343	**0.001**
Age	2	16.369	8.1843	0.15589	2.1550	1.8663	**0.036**
Site:Age	2	9.767	4.8836	0.09302	1.2859	0.6499	**0.262**
Residuals	15	56.967	3.7978	0.54255			
Total	20						

A PTA describes phenotypic changes in terms of length, angle and shape as a complementary tool to visualize and analyse the results of multivariate, factorial linear models (Adams and Collyer, 2009). The length of a trajectory describes the amount of phenotypic change of a group (e.g. site) associated with a change in ecological/evolutionary factors (e.g. age). The direction of a trajectory describes the covariation of phenotypic variables (principal components) associated with a change in an ecological/evolutionary factor (Adams and Collyer, 2009). Changes in trajectory shape occur when phenotypic changes, across factors, are accelerated or decelerated in one group relative to another, or are orientated in different directions, or both, in one or multiple portions of the trajectories (Collyer and Adams, 2013). A change in trajectory shape would mean that phenotypic changes are happening over a specific time (Collyer and Adams, 2013), i.e. in a given age class, in our case. We also calculated three aspects of the PTAs: magnitude difference (Δ*d*), trajectory correlations (TC, *θ*) and shape difference (SD, *D*_p_). Magnitude difference measures the difference in path lengths of trajectories, TC is the angular differences between trajectory principal components and SD is the square root of summed squared point differences, after scaling, centering and rotating trajectories (Collyer and Adams, 2018). Analysis was implemented in the R package RRPP (Collyer and Adams, 2018).

To test the effect of site (factor with two levels) and age (ordered factor with three levels) on the total number of erythrocyte nuclear abnormalities (count response variable), we fitted a Poisson Generalized Linear model (estimated using Maximum Likelihood), estimating the factor interactions. Residual diagnosis was conducted with R package DHARMa (Hartig, 2022) and performance (Lüdecke *et al.,* 2021). Complementarily, we used hierarchical partitioning to calculate the individual Nagelkerke’s marginal R^2^ that quantifies the relative importance of each predictor to the total marginal R^2^ (Lai *et al.,* 2022). All analyses were done in the R software v. 4.3.0 (R Core Team, 2023).

## Results

**Figure 2:**
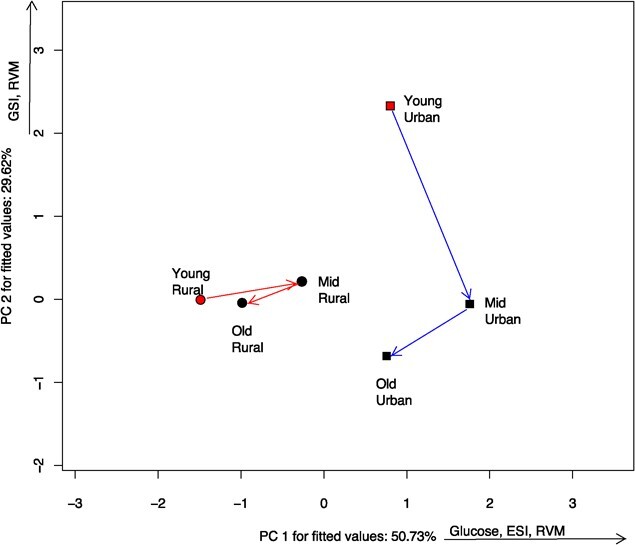
Ordination diagram showing the phenotypic trajectories for somatic indices of two populations of *Leptodactylus podicipinus*, showing the centroids of each age class and site. Age was divided into three classes: Young (1–2 LAGs), Middle age (3–4 LAGs), and Old animals (5–6 LAGs). Notice that the somatic indices of the two populations occupy distinct locations in the ordination space and change throughout adult life span in distinct directions depending on the site. While there is a strong decrease in the GSI and RVM throughout age classes in the urban population, the same is not observed in the rural counterparts. Variables shown beside axes have correlation > 0.7. ESI = Splenosomatic; GSI = Gonadosomatic Index; RVM = Relative Ventricle Mass.

We found clear differences in the location of rural versus urban populations in the multivariate space for somatic indices (Table 1). Interestingly though, the phenotypic trajectory between the two populations throughout adult life span was similar. There was no difference in length (Table 2), with the somatic indices of the urban population changing slightly more (d = 4.73) throughout their life span, than the rural one (d = 3.28). Neither angle nor shape changed significantly (Table 2). However, the variables that changed the most throughout the life span are different in each population (Fig. 2): while the urban population changed along PC2 (positively correlated with gonadosomatic index, and negatively with glucose), the indices of the rural population changed mostly along PC1 (positively correlated with splenosomatic index and glucose). Urban frogs had higher ESI, suggesting an increased immune function, and higher relative ventricular mass (RVM), suggesting higher aerobic capacity.

**Table 2:** Results of the phenotypic trajectory analysis for each phenotypic compartment, showing respective statistics. Values in bold indicate *P* < 0.05

	Correlation between trajectories (*r*)	Angle (*θ*)	Length (Δ*d*)	Shape (*D_p_*)
Somatic indices	−0.15	81.09°	1.448	0.41
Erythrocyte morphometrics	**- 0.88**	**151.13°**	0.333	0.50
Leukocyte composition	0.31	71.90°	0.325	0.5
Germ cell density	**- 0.846**	**147.83°**	**1.417**	0.359

**Figure 3:**
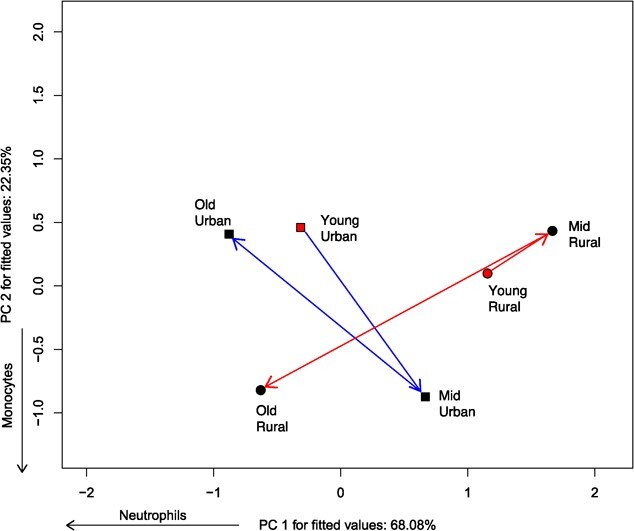
Ordination diagram showing the phenotypic trajectories for leukocyte profile of two populations of *Leptodactylus podicipinus*, showing the centroids of each age class and site. Age was divided into three classes: Young (1–2 LAGs), Middle age (3–4 LAGs), and Old animals (5–6 LAGs). The two populations follow distinct trajectories throughout their adult life span. For example, old frogs from both the rural and urban site had high neutrophil counts, whereas young rural frogs had low neutrophils. Variables shown beside axes have correlation > 0.7.

For leukocytes, we found difference in location (Table 1), but not in any aspect of the trajectory, be it length, angle or shape (Table 2). This means that the leukocyte profile of the two populations was different but changed similarly throughout the lifespan. However, there is a slight difference between populations in the cell types that most changed throughout the life span (Fig. 3). While the leukocyte profile of the rural population varied solely along PC1 (negatively correlated with neutrophils), that of the urban population changed along both PC1 and PC2 (negatively correlated with monocytes). Specifically, old individuals from both the rural and urban population had high proportion of neutrophils. But surprisingly, young individuals from urban sites also had high neutrophils.

**Figure 4:**
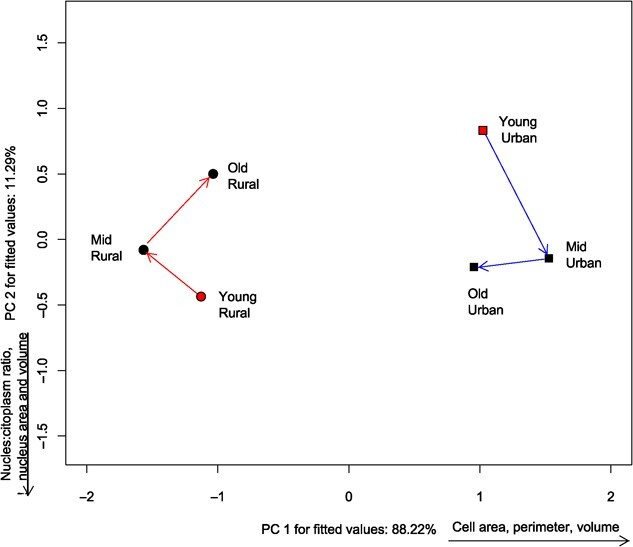
Ordination diagram showing the phenotypic trajectories for erythrocyte morphometry measured in two populations of *Leptodactylus podicipinus*, showing the centroids of each age class and site. Age was divided into three classes: Young (1–2 LAGs), Middle age (3–4 LAGs), and Old animals (5–6 LAGs). Notice that the starting points of the phenotypic trajectory of the two populations are in opposite locations in the ordination space. This means that cell morphometrics change in different directions throughout life span in the two populations. Variables shown beside axes have correlation > 0.5.

For erythrocytes, we found differences in the location (Table 1) and angles, but not in length or shape of the trajectories (Table 2). This means that not only the mean erythrocyte morphometrics is different between populations, but also that they change in different directions throughout lifespan (Fig. 4). However, the erythrocyte morphometrics vary consistently throughout life span in the two populations, that is, the amount of change is similar. Also, while the urban population had higher erythrocytes area, perimeter, and volume (positive side of PC1), the rural population had the opposite pattern (located in the negative side of PC1).

**Figure 5:**
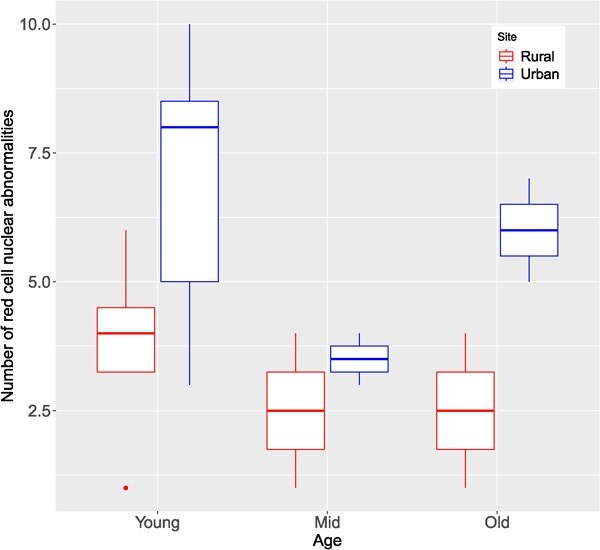
Boxplot showing the distribution of the data for erythrocyte nuclear abnormalities in the rural and urban populations of *Leptodactylus podicipinus* across age class.

Erythrocyte nuclear abnormalities were affected by site (*F*_1,15_ = 9.3432; *P* = 0.007), but not age (F_1,15_ = 2.1455; *P* = 0.151; Fig. 5). The model explained a substantial amount of variance (Nagelkerke's R^2^ = 0.67), with site individually explaining 62.63% of the variance, while age explained 37.37% (see Franco-Belussi *et al.,* 2023). The overall mean (±SD) of abnormalities in the urban (6.09 ± 2.47) was twice larger than in the rural population (3 ± 1.70). Young frogs from the urban area (6.86 ± 2.61) had two times more abnormal nuclei than rural ones (3.75 ± 2.06), while old urban frogs (6 ± 1.41) had three times more abnormal nuclei than rural ones (2.5 ± 1.29), but there was no difference in mid ages (Fig. 5).

**Figure 6:**
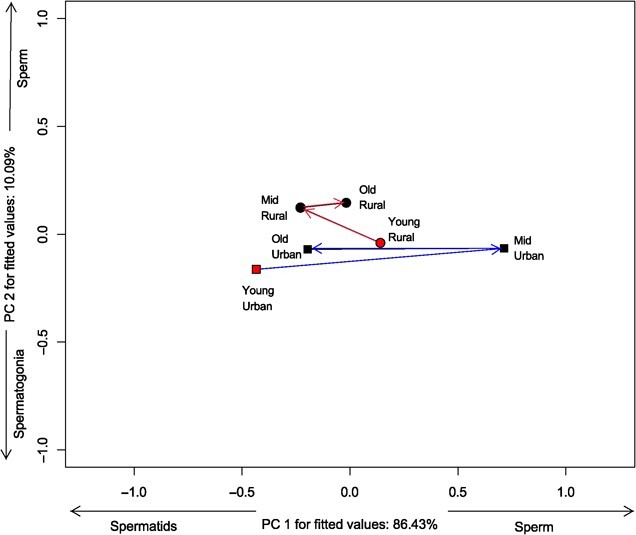
Ordination diagram showing the phenotypic trajectories for germ cell density of two populations of *Leptodactylus podicipinus*, showing centroids of each age class and site. Age was divided into three classes: Young (1–2 LAGs), Middle age (3–4 LAGs), and Old animals (5–6 LAGs). Notice the difference in length and angles between trajectories, meaning that the amount of phenotypic change in populations is different, as well as the direction of this change. Variables shown beside axes have correlation > 0.55.

For germ cell density, we found differences in location (Table 1), length and angle, but not shape (Table 2). This means that the amount of change in the density of the distinct germ cell types differs between populations throughout adult life span, with the urban population (d = 2.07) changing much more, than the rural one (d = 0.65). Also, the germ cell composition of the two populations occupies distinct locations in the ordination space (Fig. 6), meaning that they have different means of cell type densities. It is noteworthy that germ cell density of the urban population changes more along the PC1 (positively correlated with sperm, and negatively with spermatids), with both older and younger frogs having more spermatids, whereas middle-aged animals have more sperm. The pattern seems to be opposite for the rural population, but it is far less strong (Fig. 6). This is consistent with the strong negative correlation (*r* = − 0.846) between angles of trajectories (Table 2), meaning that germ cell densities of the two populations indeed follow different paths throughout adult life span.

## Discussion

Taken together, our results demonstrate that urban populations start reproducing earlier, had a higher metabolic rate, inferred from high blood glucose and smaller erythrocyte nucleus/cytoplasm ratio, more erythrocyte nuclear abnormalities, and an altered leukocyte profile. This is the first study providing evidence that urban lifestyle produces consistent changes in multiple morphophysiological parameters in a vertebrate in a tropical city from the Global South.

Somatic indices and glucose levels demonstrated that urban individuals get old and become less healthy faster than those the rural ones. It also suggests a higher basal metabolic rate in urban, than in the rural population. Site explained slightly higher proportion of variance (semi-partial R^2^ = 0.20) than age (semi-partial R^2^ = 0.15). Urban frogs had higher relative ventricular mass, which is positively related to dehydration tolerance in several frog lineages (Withers and Hillman, 2001). Thus, a high RVM in urban frogs might suggest they have high tolerance to dehydration due a cardiovascular resistance to homeostatic imbalance. Urban frogs had also higher ESI, suggesting an increased immune function. Urban frog population may have higher levels of glucose than rural ones due to a variety of factors. One possible explanation is that urban environments may have higher levels of pollution and contaminants, which can increase oxidative stress and damage to cells, including erythrocytes. In addition, urban environments usually have higher temperatures (Diamond and Martin, 2020) (so-called urban heat island), which can also increase glucose levels in frogs (Farrar and Frye, 1979, Tindal, 1956). High temperatures can lead to dehydration and increased metabolic demands (Rollins-Smith and Woodhams, 2011), which can increase glucose production to provide energy for physiological processes (Hillman *et al.,* 2008). Lastly, exposure to noise and light pollution and other sources of chronic stress in urban environments can also lead to an increase in glucose levels in frogs. Chronic stress can activate the hypothalamic–pituitary–adrenal (HPA) axis, increasing glucocorticoid hormones that augment glucose production (Rollins-Smith and Woodhams, 2011; Gomes *et al.,* 2022).

The mean leukocyte profile was different between rural and urban populations (partial R^2^ = 0.10), but age seemed a more important factor (partial R^2^ = 0.21). This is still consistent with PoLS, given that immune capacity of individuals decreases with age, but that of urban population seems to decrease faster. The main cell types with high loadings in the first two PCs were monocytes and neutrophils. Both cells modulate innate immune responses (Rollins-Smith and Woodhams, 2011). Interestingly, lymphocytes, and not monocytes and neutrophils had the highest counts in both males (this study) and females (Franco-Belussi *et al.,* 2022) of this species. This means that lymphocytes, although prevalent, are not important to differentiate the two populations. We found that old individuals from both the rural and urban population had high neutrophil counts. But surprisingly, young urban frogs also had high neutrophils. The immune response of monocytes is more specialized, intense, but more costly than neutrophils. This conforms to the idea that young individuals prioritize resistance over tolerance (Schoenle *et al.,* 2018). Additionally, this leukocyte profile pattern suggests that urban frogs are in contact with stressors since young age, which would explain why monocytes are more activated or proliferated, than in young rural frogs, which possibly reflect the normal physiological conditions for the species. Alternatively, while we did not collect data on advertisement call in our study sites, an increased calling rate in urban frogs would increase the corticosterone levels and consequently lead to a higher neutrophil count. There is no consensus that urbanization increases male calling rate. In a recent review of experimental and observational studies, 22 of them found a decrease in calling rate, while 20 found no change, and an increase in 11 studies in several frog species around the world (reviewed in Zaffaroni-Caorsi et al., 2022). Previous studies found little effect of urban habitats on immune response of frogs (Iglesias-Carrasco *et al.,* 2017) and birds (Cavalli *et al.,* 2018). However, other studies found a high heterophil/lymphocyte ratio in urban birds (Ribeiro *et al.,* 2022), indicating they have an altered leukocyte profile caused by chronic stress. Our three urban sites are along a stream that is constantly polluted by sewage and siltation (Diniz *et al.,* 2021), promoting eutrophication. Acoustic pollution and artificial light at night are also high in those sites. Therefore, we would expect to find a stronger effect of urban versus rural contrast on leukocytes. However, our ability to infer immune responses from leukocyte count alone is limited (Davis *et al.,* 2008; Zhelev and Popgeorgiev, 2021) and more refined data may be necessary to capture frog responses to environmental change.

Concurrently, aging seems to be a stronger factor determining leukocyte profile in our frog populations. There are a few data on the immunosenescence in wild frogs, especially tropical species and based on circulating leukocytes, with most studies (Torroba and Zapata, 2003; Müller *et al.,* 2013) comparing larvae and adults and examining specific glandules, like the thymus. Our results agree with previous papers showing that short-lived amphibians, like frogs, show gradual senescence (Torroba and Zapata, 2003). Taken together, populations seem to follow divergent life-history strategies (Ricklefs and Wikelski, 2002; Rollins-Smith and Woodhams, 2011): while the urban population seems to trade-off immunological resistance for high reproductive output, the rural population preserves its immune tolerance while reproducing at latter ages with less intensity. However, the functional aspects of immunological cells remain poorly understood in frogs, compared to mammals. As such, the phagocytic ability of neutrophils in frogs and the cellular cooperation between lymphocyte and monocytes is not yet fully described. Despite previous studies have examined immunosenescence in birds, our study is one of the few that examined one aspect of immunosenescence (circulating leukocytes) throughout the adult life span of a vertebrate in a spatial context (Becker *et al.,* 2020), across a putative stress gradient. This spatial pattern we found in immune defense seems to be derived from the physiological challenges associated with urbanization. Further experimental studies that explicitly manipulate UV radiation, temperature, and photoperiod throughout the adult lifespan are needed to investigate the mechanism involved in changes we found in the leukocyte profile in frogs.

Erythrocyte morphometrics indicated that urban frogs had in mean larger cells that were more active, than those in the rural area. Site explained a much larger variance in cell shape (semi-partial R^2^ = 0.18) than age (semi-partial R^2^ = 0.01). Urban frogs might develop larger erythrocytes compared to rural populations due to exposure to environmental stressors, such as pollution. In addition, some studies have shown that exposure to heavy metals (Marques *et al.,* 2009) and pesticides (Gavel *et al.,* 2021) damage erythrocytes. Larger cells with larger nuclei are younger and more frequently renovated. Conversely, the chromatin is more condensed in smaller nuclei, indicating that these are older cells (Rothmann *et al.,* 2000). There is some evidence that oxidative stress damages cell membrane, diminishing erythrocyte deformability, making them less efficient in delivering oxygen to tissues (Mohanty *et al.,* 2014). We also found that urban individuals had more erythrocyte nuclear abnormalities than rural ones, regardless of age, but this effect was more pronounced at early and latter ages, than mid ones. Young individuals usually have a high cell turnover than older ones (Forman and Just, 1976). For example, nuclear abnormalities were reversed in tadpoles of *Boana pulchella* exposed to an herbicide as they developed (Perez-Iglesias *et al.,* 2018). An increase in nuclear abnormalities in older individuals can also represent the cumulative effects of xenobiotics in the organism (Romanova *et al.,* 2021). Therefore, the larger nucleus volume in old urban frogs suggests that the chromatin is less compacted (Hübner and Spector, 2010), but this is likely due to a high number of cell abnormalities they experience. Our results also provide an important finding that age must explicitly be considered when comparing nuclear abnormalities between areas. This has profound consequences for ecotoxicological studies comparing disturbed sites with reference ones.

We found that germ cells composition was different between age groups and sites, with age having a slightly stronger influence (semi-partial R^2^ = 0.01) than site (semi-partial R^2^ = 0.004). Our results suggest that individuals from the urban population reach an older reproductive status more quickly, than those from the rural population. Consequently, rural individuals are more likely to continue reproducing at latter ages, than urban ones. These age-related differences between sites may reflect distinct selection pressures or constraints associated with environmental conditions or stressors, which could contribute to variations in the duration of the breeding season at each site. In captive male frogs, the lack of seasonal cues reduces circulating testosterone, resulting in lower sperm production (Julien et al., 2022). Urban environments pose several challenges to frog reproduction, but changes in germ cells may not necessarily be related to pollutants (Birnie-Gauvin *et al.,* 2016). One factor that affects frog reproduction in urban environments is anthropogenic noise, which increases circulating corticosterone levels and reduces sperm count and viability (Kaiser *et al.,* 2015). The underlying process for this decrease is related to the disruption of the hormonal reproductive axis caused by stress (Kaiser *et al.,* 2015). However, previous studies comparing germ cell density between contrasting environments did not consider animal's age. As such, it remains unclear if the effects are due to a specific or a combination of environmental stressors (e.g. temperature, photoperiod, rainfall, xenobiotics), or to natural reproductive senescence. Here, we found that the quantity of sperm was higher in mid-aged urban frogs, while spermatid density was higher in young urban frogs. Thus, urban frogs might initiate reproduction at a younger age, compared to their rural counterparts. Future studies could, for example, evaluate how the incidence of light and xenobiotics interfere with the production of secondary sexual hormones in frogs.

Overall, our results demonstrate that the traits we evaluated were highly plastic in a way that is consistent with a faster pace-of-life in urban frogs compared to rural ones. However, except for nuclear abnormalities, most of the responses were non-parallel (Bolnick *et al.,* 2018), indicating that populations in each area displayed unique trajectories in different phenotypic aspects throughout their lifespan. Although age was more important than the rural–urban contrast in determining the leukocyte profile, data on germ cell density suggest that urban male frogs might be trading-off immunological resistance for reproductive output, especially at early ages. This is consistent with a recent meta-analysis (Foo *et al.,* 2023) that highlights the significance of age class in shaping immune investment. Assuming that the rural population represents a stock closer to the ancestral population that colonized the urban area, it seems that different compartments of the phenotype have evolved towards distinct optima in response to adaptation to urban life. Interestingly, these effects have been already reported for other vertebrates inhabiting temperate zones (Birnie-Gauvin *et al.,* 2016; Murray *et al.,* 2019), but this is the first study to demonstrate similar effects in tropical zones. Urban areas in the Global North have a much prolonged and slower urbanization rate than those in the Global South (McHale *et al.,* 2013; Shackleton *et al.,* 2021). Our results provide a first step towards understanding the efficiency of morphophysiological variables to provide a more comprehensive assessment of wild frog population health and viability, especially in response to urbanization. Somatic indices and erythrocyte nuclear abnormalities appear to be especially useful to detect the effects of multiple stressors associated with urbanization on organismal physiology. Future studies should employ experimental approaches to unravel the mechanisms involved in phenotypic changes related to the ability of frogs to live in urban areas.

## Supplementary Material

Web_Material_coad106
